# Comparative Evaluation of the Effect of Different Irrigation Regimens With and Without Ultrasonic Activation on Root Dentin Microhardness: An In Vitro Study

**DOI:** 10.7759/cureus.73854

**Published:** 2024-11-17

**Authors:** Sergy A, Navjot Khurana, Jagvinder Mann, Arshdeep Kaur, Naveen Prasath, Jaspreet Kaur

**Affiliations:** 1 Conservative Dentistry and Endodontics, Government Dental College and Hospital, Patiala, IND

**Keywords:** chlorhexidine, dentin, microhardness, sodium hypochlorite, ultrasonic activation

## Abstract

Background

The structural properties of dentin, including its microhardness, can be affected by exposure to endodontic irrigating solutions and ultrasonic activation.

Aim

The current study aimed to assess the effect of three different irrigation regimens, with and without ultrasonic activation, on the microhardness of the dentin.

Methodology

The research was conducted on 120 mandibular premolars randomly divided into four groups (n=30) based on the irrigation regimen, with each group further divided into two subgroups (n=15). Subgroups ‘a” were subjected to respective irrigation regimens without ultrasonic activation. Subgroups ‘b’ were subjected to ultrasonic activation. The Vickers microhardness test was conducted on the samples, and the data underwent statistical analysis using the independent sample t-test, ANOVA, and the Tukey test.

Results

The observations of the study unveiled that the microhardness of the root dentin was decreased by all the irrigation regimens. Microhardness values of dentin showed higher reduction with ultrasonic activation than without ultrasonic for all groups.

Conclusions

The dentin microhardness was significantly reduced when 3% and 5% sodium hypochlorite were used with 17% ethylenediaminetetraacetic acid (EDTA) as irrigation solutions. Ultrasonic activation of the irrigants had an adverse effect on the microhardness of dentin.

## Introduction

The effectiveness of endodontic treatment relies on the eradication of the existing microbes within in the root canal and preventing their regrowth. Removal of debris, biofilm, necrotic tissues, and microbes found within the root canal is performed manually or through rotary shaping, as well as frequent and copious canal irrigation [1].

Current endodontic therapy uses irrigants such as sodium hypochlorite (NaOCl), ethylenediaminetetraacetic acid (EDTA), and chlorhexidine gluconate (CHX). Unfortunately, there is no single irrigating solution available till date that fulfills all these ideal requisites [1].

NaOCl is commonly employed as an irrigation solution for root canals in endodontic procedures. Its primary function is to eliminate bacteria that form biofilms, fungi, and viruses. Aside from its exceptional antibacterial qualities, NaOCl also possesses the capability to break down residual pulp tissues and the organic constituents of dentin through a non-specific proteolytic action. However, it does not impact the inorganic portion of dentin [2].

EDTA is the most frequently employed irrigating solution to eliminate the inorganic residues not removed by NaOCl. Even though EDTA has no antibacterial properties, it can get rid of the smear layer deposited by the mechanical action of instruments, making the canal walls accessible to sealer [3].

CHX is commonly recommended as an exceptional root canal irrigant owing to its low toxicity, substantivity, and wide spectrum of antimicrobial action especially against *Staphylococcus aureus*, *Candida albicans*, *Enterococcus faecalis*, *Porphyromonas endodontalis*, *Porphyromonas gingivalis*, and *Prevotella intermedia*. Nevertheless, the inability of CHX to dissolve tissues has been identified as its primary drawback [4].

Ultrasonic activation has emerged as predominant method for activating irrigants in root canals. Rather than directly impacting the main canal, ultrasonic files primarily work by acoustic streaming the surrounding irrigant. This process helps the irrigant reach all regions of the root canals, improving mechanical cleaning, enhancing the disinfectant action, and increasing wall shear stress [5].

Microhardness is regarded as an indirect indication of alterations in mineral changes in root canal dentin; the changes could potentially affect the bonding properties of the dentin surface. Microhardness serves as an indicator of mineral changes, potentially impacting the adhesion capabilities of dentin surfaces [6].

The impact of a combination of irrigating solutions and chelating compounds on dentin should be tested as they might interact with each other during the procedure. Many studies have examined the effects of irrigants on root dentin microhardness, but more research is needed on various irrigation protocols with chelating agents, both with and without ultrasonic activation.

The current research aimed to assess the influence of three different irrigation regimens, with and without ultrasonic activation, on the microhardness of root canal dentin.

## Materials and methods

The Institutional Review Board of Government Dental College and Hospital, Patiala, Punjab, India, approved the research proposal, and ethical clearance was obtained (Ref no: CONS/2/2024). Based on the results from a previous study conducted by Pascon et al. [7], the present research calculated the sample size using G*Power (RRID: SCR_013726) to identify differences among the four groups. The calculations aimed for 80% power, an effect size of 0.74, and a significance level of 0.05, leading to a recommendation of at least 30 samples per group. The study was conducted in the Department of Conservative Dentistry and Endodontics, Government Dental College and Hospital, Patiala from the beginning of January 2023 until the end of January 2024.

Teeth selection

The research was conducted on 120 unrestored mandibular premolars extracted due to periodontal issues and orthodontic treatment gathered from the Department of OMFS (Oral and Maxillofacial Surgery), Government Dental College and Hospital, Patiala. Teeth with mature apices, single patent canals (Vertucci’s type I), intact root surface, and devoid of external defects or cracks were included. Teeth with open apices, severely curved root canals, damaged root surfaces, anatomical variations, and root caries were excluded.

Preparation of samples

All teeth were cleaned thoroughly to remove any soft tissue, debris, or calculus deposition using periodontal curette. The teeth were kept hydrated during the study by storing them in distilled water at room temperature. A diagnostic radiograph of each tooth was taken from the proximal direction to confirm the existence of a single patent canal.

Mounting of specimen

**Figure 1:**
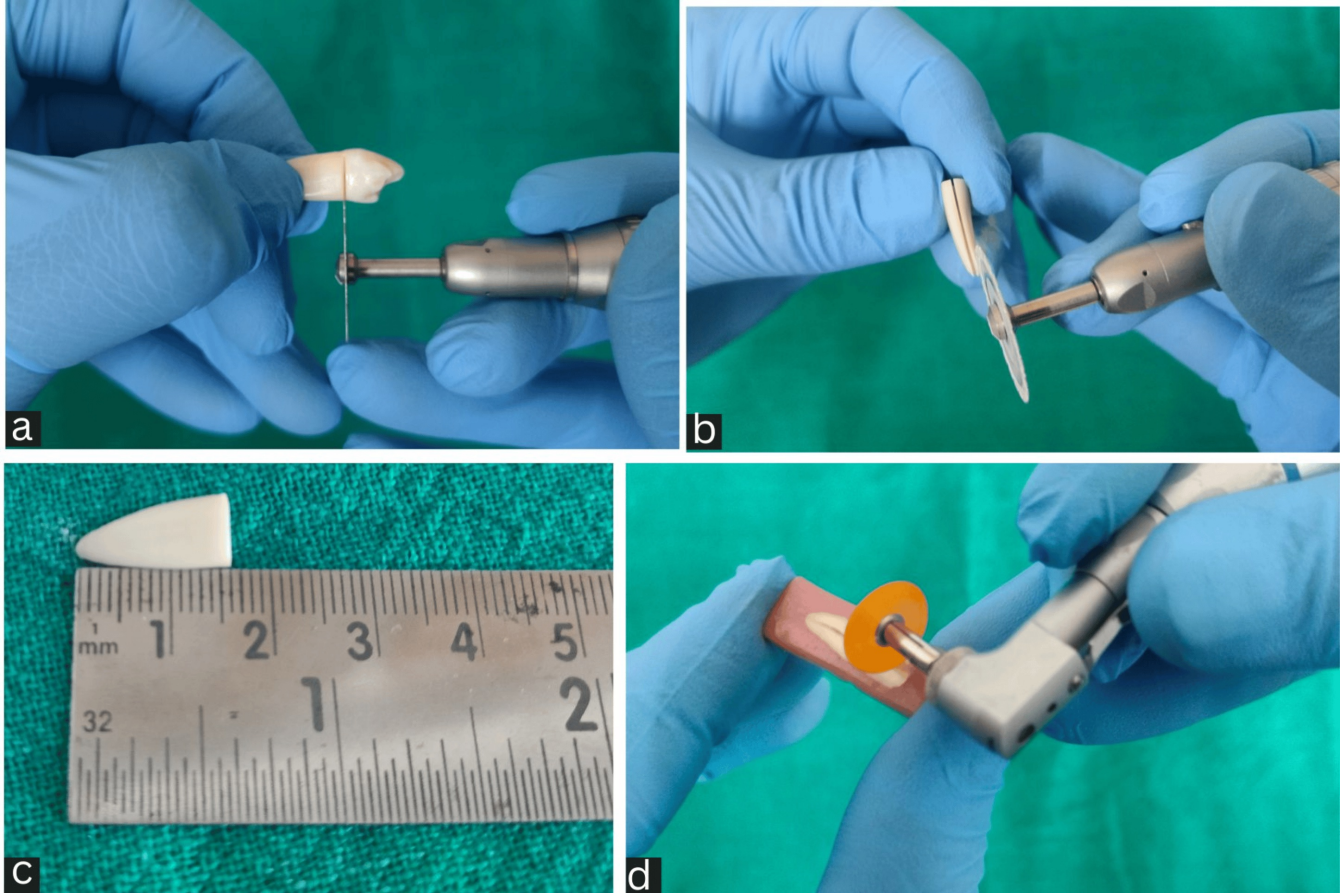
(a) Decoronation at the cementoenamel junction. (b) Sectioning in a buccolingual direction. (c) The roots measured at a length of 16±1 mm. (d) Root halves were polished with polishing discs.

The teeth underwent decoronation at the cementoenamel junction (Figure 1a) with a diamond disc while being cooled with water, resulting in the roots being left at a length of 16 ± 1 mm (Figure 1c). The roots were then sectioned along the long axis in a buccolingual direction (Figure 1b) with a low-speed diamond disc. Each root was bisected, encased in acrylic resin with the dentin surface exposed, and polished using 500- and 600-grit sandpapers, followed by finer discs (Sof-Lex, 3M ESPE, Salt Lake City, UT, USA) (Figure 1d) in the following order: finish (fine: 3-9 µm) and polish (superfine: 1-7 µm) under copious water to avoid heat generation and debris accumulation.

Groups

The assorted specimens were randomly divided into the following four test groups, containing 30 samples each depending on the irrigant solution they were immersed in (Table 1):

Group 1: 50 mL of distilled water for 5 minutes (control group)

Group 2: 50 mL of 3% NaOCl (Prevest DenPro, Jammu City, Jammu and Kashmir, India) and 50 mL of 17% EDTA (Prevest DenPro) for 5 minutes each

Group 3: 50 mL of 5% NaOCl (Ammdent, Mohali, Punjab, India) and 50 mL of 17% EDTA (Prevest DenPro) for 5 minutes each

Group 4: 50 mL of 2% CHX (Prevest DenPro) and 50 mL of 17% EDTA (Prevest DenPro) for 5 minutes each

**Table 1:** Groups according to the irrigation regimen and ultrasonic activation

Groups	Irrigation regimen
Group 1	Distilled water
Group 2	3% NaOCl +17% EDTA
Group 3	5% NaOCl +17% EDTA
Group 4	2% CHX +17% EDTA
Subgroups	
a	Without ultrasonic activation
b	With ultrasonic activation

Division into subgroups

All groups were segregated into two subgroups of 15 specimens each. Subgroups1a, 2a, 3a, and 4a were subjected to respective irrigation regimens without ultrasonic activation. Subgroups 1b, 2b, 3b, and 4b were subjected to ultrasonic activation (Woodpecker Medical Instrument Co., Ltd Guilin, Guangxi, China) at 42,000 Hz frequency for 2 minutes.

All specimens in groups 2, 3, and 4 were irrigated with 50 mL of distilled water after immersion in each solution (NaOCl, CHX, and EDTA) to eradicate the chemical interaction among the substances and prevent the extended impact of the chelating agent. After the surface treatment, absorbent paper was used for sample drying.

Microhardness testing

**Figure 2:**
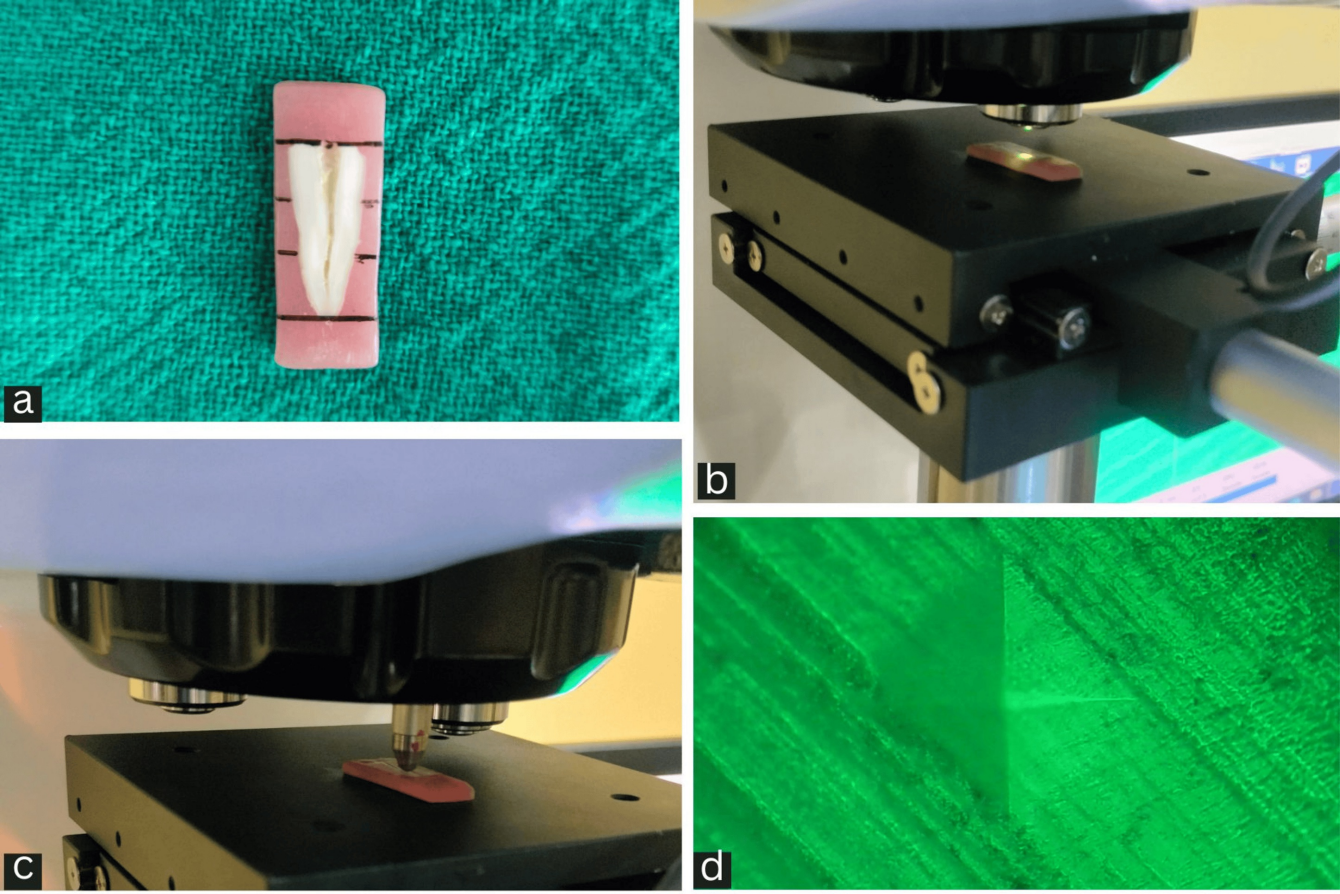
(a) Coronal, middle, and apical divisions of sample. (b) Sample was examined under a microscope attached with the Vickers hardness tester at 40x. (c) Indentation made perpendicular to the root surface. (d) Microscopic image of Vickers indentation produced on specimens.

The specimens were air-dried on absorbent paper before testing on the Vickers microhardness platform (Model: ASI-HMVT, A. S. I. Sales Private Limited, New Delhi, India). The canal lumen was segmented into three distinct segments on both sides - coronal part, middle part, and apical part - for each root half (Figure 2a). The samples were analyzed in detail under a microscope attached with the Vickers hardness tester at 40x magnification (Figure 2b). Indentations were made on the dentinal surfaces of each sample's coronal, middle, and apical regions, 500 micrometers from the pulp-dentin junction and perpendicular to the root surface, using 300 g of load maintaining for 20 seconds (Figure 2c). The diagonal dimensions of the indentations (Figure 2d) were evaluated and converted automatically into Vickers hardness number (VHN) values by a built-in software (hardness software measure GB/T 4340.1-2009, BradyKnows Medical, Beijing, China) for image analysis. The VHN values were transformed and presented on the device's monitor. The final microhardness value of each specimen was obtained by calculating the mean of the three recorded values.

Statistical analysis

The obtained data were analyzed for intergroup comparison using the statistical package SPSS Version 26.0 (IBM Corp., Armonk, NY), and the level of significance was set at p<0.05. The data underwent statistical analysis using the independent sample t-test, ANOVA, and the Tukey test. The confidence level was maintained at 95% (p<0.05).

Statistical analysis was conducted on the collected data. One-sample Kolmogorov-Smirnov test was used, which showed that the data were normally distributed. Since the data were normally distributed, the independent sample t-test was applied to compare the values of microhardness of subgroup a (without ultrasonic activation) and subgroup b (with ultrasonic activation).

 Since the data were normally distributed, the one-way ANOVA test was applied. The Tukey test was employed for intergroup comparison.

## Results

Results of the study showed that different irrigation regimens reduced the microhardness of the root dentin. Microhardness values of dentin showed higher reduction with ultrasonic than without ultrasonic for all groups (p>0.05) (Table 2). Samples in group 4a (2% CHX and 17% EDTA) showed the lowest reduction compared to group 1a (control group), and the difference was determined to be highly statistically significant (p<0.001) (Table 3). Samples in group 3b (5% NaOCl and 17% EDTA) showed the highest reduction compared to group 1b (control group), and the difference was statistically highly significant (p<0.001).

**Table 2:** Independent samples test and statistical analysis of mean microhardness and its standard deviation of groups without and with ultrasonic activation between all four groups NaOCl, sodium hypochlorite; EDTA, ethylenediaminetetraacetic acid; CHX, chlorhexidine gluconate

Groups	N	Without ultrasonic (subgroup a)			With ultrasonic (subgroup b)			Independent samples test	
		Minimum	Maximum	Mean±SD	Minimum	Maximum	Mean±SD	t-Value	p-value
Group 1 (distilled water)	15	57.20	66.52	62.235±2.958	56.54	66.30	60.713±2.899	1.423	0.166
Group 2 (3% NaOCl + 17% EDTA),	15	46.39	51.34	48.783±1.779	43.37	51.55	48.392±1.985	0.568	0.575
Group 3 (5% NaOCl + 17% EDTA),	15	45.07	50.10	47.039±1.584	42.19	49.57	46.269±2.109	1.130	0.268
Group 4 (2% CHX + 17% EDTA)	15	47.93	54.94	51.700±2.082	47.19	53.73	50.581±2.073	1.473	0.152

**Table 3:** One-way ANOVA and multiple comparisons by Tukey test in all experimental and control groups with and without ultrasonic activation p<0.001 was considered as highly significant (HS), p<0.05 was considered as significant (S), and p>0.05 was considered as non-significant (NS).

Groups	Comparison groups	Tukey				One-way ANOVA		Significance
		Mean difference	p-Value	Lower bound	Upper bound	df	f-Value	
Group 1a	Group 2a	13.45195	<0.001	11.3574	15.5465	3	148.13	HS
	Group 3a	15.19627	<0.001	13.1017	17.2908			HS
	Group 4a	10.53563	<0.001	8.4411	12.6302			HS
Group 2a	Group 1a	-13.45195	<0.001	-15.5465	-11.3574			HS
	Group 3a	1.74432	>0.05	-0.3502	3.8388			NS
	Group 4a	-2.91633	>0.05	-5.0109	-0.8218			HS
Group 3a	Group 1a	-15.19627	<0.001	-17.2908	-13.1017			HS
	Group 2a	-1.74432	>0.05	-3.8388	0.3502			NS
	Group 4a	-4.66064	<0.001	-6.7552	-2.5661			HS
Group 4a	Group 1a	-10.53563	<0.001	-12.6302	-8.4411			HS
	Group 2a	2.91633	<0.05	0.8218	5.0109			S
	Group 3a	4.66064	<0.001	2.5661	6.7552			HS
Group 1b	Group 2b	12.32105	<0.001	10.1004	14.5417	3	116.34	HS
	Group 3b	14.44434	<0.001	12.2237	16.6650			HS
	Group 4b	10.13189	<0.001	7.9112	12.3526			HS
Group 2b	Group 1b	-12.32105	<0.001	-14.5417	-10.1004			HS
	Group 3b	2.12329	>0.05	-0.0974	4.3440			NS
	Group 4b	-2.18916	<0.05	-4.4098	0.0315			S
Group 3b	Group 1b	-14.44434	<0.001	-16.6650	-12.2237			HS
	Group 2b	-2.12329	>0.05	-4.3440	0.0974			NS
	Group 4a	-4.31245	<0.001	-6.5331	-2.0918			HS
Group 4b	Group 1b	-10.13189	<0.001	-12.3526	-7.9112			HS
	Group 2b	2.18916	>0.05	-0.0315	4.4098			NS
	Group 3b	4.31245	<0.001	2.0918	6.5331			HS

## Discussion

The goal of root canal therapy is to eliminate microorganisms from the root canal using irrigating solutions. In complex teeth, effective chemical debridement is essential, necessitating a strategic combination of irrigants applied in the correct sequence for success [8].

In the present study, the microhardness of radicular dentin was significantly reduced when 3% and 5% NaOCl with 17% EDTA were used as endodontic irrigants. This is attributed to the organic solubilizing characteristics of NaOCl on the collagen part of dentin [9]. The findings of the present research align with the earlier research conducted by Ari et al. and Oliveira et al., who determined that NaOCl plays a crucial role in diminishing the microhardness of dentin within the root canal [9,10]. Kinney et al. showed that reduced hardness is linked to decreased rigidity in the intertubular dentin matrix, which is caused by uneven mineral distribution within the collagen matrix [11]. A noteworthy discovery revealed that the reduction in microhardness after 5% NaOCl irrigation did not show a statistically significant variance compared to the effects seen with 3% NaOCl.

The application of NaOCl subsequent to the chelator resulted in the degradation of collagen that had been exposed by the chelating agent during the final phase, which may contribute to a more significant reduction in hardness and an exacerbation of erosion [12]. In our study, we used 3% and 5% NaOCl before applying 17% EDTA. Qian et al. found that NaOCl prior to EDTA reduces the average size of dentinal tubule openings, affecting dentinal erosion [13].

EDTA has the capability to decalcify root dentin up to a depth of 20-50 μm with an application time of 2 to 3 minutes [14]. De-Deus et al. studied the effect of 17% EDTA on radicular dentin microhardness over 1, 3, and 5 minutes, finding the most significant decrease between baseline and 3 minutes, with stabilization at 5 minutes [15].

Oliveira et al. found that a 2% chlorhexidine solution significantly reduced the microhardness of root dentin at 500-µm and 1,000-µm depths [10]. In contrast, our study showed that the microhardness reduction in the 2% CHX group was similar to the control group, with no significant difference (p > 0.05). This decrease in microhardness may be linked to chlorhexidine's effect on the Ca/P ratio [10].

The review of the data illustrated that the groups subjected to ultrasonic agitation exhibited a greater decrease in microhardness in comparison to the groups that were not exposed to ultrasonic agitation. Paque et al. provided further evidence supporting the effectiveness of ultrasonic activation in the removal of hard tissue debris using NaOCl and EDTA [16]. After 30 seconds of ultrasonic activation of the irrigant without any additional supply, Cameron observed an increase in intracanal temperature ranging from 37°C to 45°C near the instrument's tip and 37°C further away from the tip, which could adversely impact the mechanical properties of dentin [17].

Our research indicate that enhanced activation of the irrigation regimen led to reduction in dentin microhardness compared to without activated groups using similar or higher concentrations of NaOCl. Therefore, the assessment of microhardness can offer indirect insights into mineral alterations within dental hard tissues.

The findings of the study can be attributed to factors such as the organic dissolving properties of NaOCl on the collagen part of dentin, which could lead to softening of dentin with the application of both 3% and 5% NaOCl. In addition, CHX has the capability to alter the Ca/P ratio, leading to a possible reason for the decreased microhardness values observed in root dentin following exposure to the group treated with 2% CHX and 17% EDTA. The acoustic streaming generated by the activation of disinfecting solutions may negatively impact the biomechanical properties of root dentin.

In this research, we aimed to identify the most deleterious impacts of irrigants and ultrasonic activation on dentin. The change in structural properties of mineralized tissues leads to a substrate that is less resilient and more prone to brittleness. This increased brittleness can make teeth that have undergone endodontic therapy more vulnerable to fractures in the crown or root. Modifications in dentin's structural properties can impact the bonding of adhesive materials at a microscopic level, which is clinically significant.

A key limitation of this study is the disparity between the research environment and clinical conditions, where factors such as blood and residual tissue may impact irrigant efficacy. In vivo studies are needed to validate the findings, and further research should explore the effects of irrigants in closed-canal systems and the use of agitation devices such as sonic and ultrasonic systems.

## Conclusions

The findings of this study showed that all irrigation regimens reduced the microhardness of the root dentin. The degree of reduction in microhardness was directly proportional to the concentration of the irrigant used. The microhardness of root dentin was significantly reduced when 3% and 5% NaOCl were used as a root canal irrigation solution. The group treated with 2% CHX also exhibited a decrease in microhardness as compared to the control group, and this difference was not statistically significant. The groups subjected to ultrasonic activation exhibited a greater reduction in microhardness when compared to groups not subjected to ultrasonic activation.
